# NIR-Sensitive Squaraine Dye—Peptide Conjugate for Trypsin Fluorogenic Detection

**DOI:** 10.3390/bios14100458

**Published:** 2024-09-25

**Authors:** Priyanka Balyan, Shekhar Gupta, Sai Kiran Mavileti, Shyam S. Pandey, Tamaki Kato

**Affiliations:** Graduate School of Life Science and System Engineering, Kyushu Institute of Technology, 2-4 Hibikino, Wakamatsu-Ku, Kitakyushu-Shi, Fukuoka 808-0196, Japan; priyanka101@mail.kyutech.jp (P.B.); gupta.shekhar932@mail.kyutech.jp (S.G.); mavileti-chilamakuru.kiran-sai678@mail.kyutech.jp (S.K.M.)

**Keywords:** trypsin detection, aggregation-caused quenching, FRET, NIR detection, squaraine dye, dye–peptide conjugate

## Abstract

Trypsin enzyme has gained recognition as a potential biomarker in several tumors, such as colorectal, gastric, and pancreatic cancer, highlighting its importance in disease diagnosis. In response to the demand for rapid, cost-effective, and real-time detection methods, we present an innovative strategy utilizing the design and synthesis of NIR-sensitive dye–peptide conjugate (**SQ-3 PC**) for the sensitive and selective monitoring of trypsin activity by fluorescence ON/OFF sensing. The current research deals with the design and synthesis of three unsymmetrical squaraine dyes **SQ-1**, **SQ-2**, and **SQ-3** along with a dye–peptide conjugate **SQ-3-PC** as a trypsin-specific probe followed by their photophysical characterizations. The absorption spectral investigation conducted on both the dye alone and its corresponding dye–peptide conjugates in water, utilizing **SQ-3** and **SQ-3 PC** respectively, reveals enhanced dye aggregation and pronounced fluorescence quenching compared to observations in DMSO solution. The absorption spectral investigation conducted on dye only and corresponding dye–peptide conjugates in water utilizing **SQ-3** and **SQ-3 PC**, respectively, reveals not only the enhanced dye aggregation but also pronounced fluorescence quenching compared to that observed in the DMSO solution. The trypsin-specific probe **SQ-3 PC** demonstrated a fluorescence quenching efficiency of 61.8% in water attributed to the combined effect of aggregation-induced quenching (AIQ) and fluorescence resonance energy transfer (FRET). FRET was found to be dominant over AIQ. The trypsin-mediated hydrolysis of **SQ-3 PC** led to a rapid and efficient recovery of quenched fluorescence (5-fold increase in 30 min). Concentration-dependent changes in the fluorescence at the emission maximum of the dyes reveal that **SQ-3 PC** works as a trypsin enzyme-specific fluorescence biosensor with linearity up to 30 nM along with the limit of detection and limit of quantification of 1.07 nM and 3.25 nM, respectively.

## 1. Introduction

Proteases are enzymes that catalyze the hydrolysis of peptide bonds, playing crucial roles in various physiological and pathological processes [1]. Among these, trypsin, a serine protease produced in the pancreas, is particularly significant due to its role in digestion and its involvement in pancreatic disorders such as pancreatitis and pancreatic cancer [2,3,4]. Trypsin facilitates the detachment of adherent cells and is therefore used therapeutically in tissue regeneration and as an anti-inflammatory agent [5]. The expression of trypsin is significantly elevated in several human cancer cells found in the stomach, colon, lung, and breast [6]. Recently, trypsin has gained recognition as a potential marker for cancer patients [7,8]. This is because the dysregulation of trypsin has been associated with the development of several tumors, such as colorectal cancer (CRC), gastric cancer, and pancreatic cancer [9,10]. In addition, trypsin is often used in the field of food chemistry for protein identification purposes [11]. In light of its biological significance, there is a significant demand for inventive and accessible assays for trypsin. These assays are essential for the development of effective diagnostic and therapeutic techniques for the treatment of pancreatic diseases and as applications in the proteomics field [12,13]. Various techniques have been developed for trypsin detection, including mass spectroscopy [14,15], gel electrophoresis [16], enzyme-linked immunosorbent assay (ELISA) [17,18], colorimetric [19,20], and fluorometric approaches [21,22]. Among these, fluorogenic sensing technologies have gained prominence due to their simplicity, accessibility, and visual detection capabilities. These methods eliminate the need for sophisticated instrumentation and complex analytical procedures, contributing to their widespread adoption in various fields of study.

Capitalizing on these advantages, researchers have developed a diverse array of sensors employing distinctive fluorescence approaches, specifically designed and validated for the precise and sensitive detection of trypsin activity [23,24]. Recent advances in protease assay methods have focused on developing immunoassays using antibodies to bind target proteases [25,26]. However, while effective for quantifying protease levels, these immunoassays are limited in their ability to map protease activity and its correlation with disease progression. To address this limitation, alternative approaches have been explored, utilizing tailored peptide substrates with optical detection techniques such as absorbance or fluorescence spectroscopy [27,28,29]. These techniques leverage protease-substrate interactions to measure enzymatic activity directly. One of the challenges in developing fluorescence-based protease assays is the interference from the autofluorescence of biological samples and potential photo-damage to biological species due to high-energy excitation. The use of fluorophores that emit in the near-infrared (NIR) wavelength region offers a solution to these issues, providing increased sensitivity and the potential for deep-tissue bio-imaging [30,31].

In this context, NIR-sensitive squaraine dyes have emerged as promising candidates for biosensing applications due to their exceptional physical and chemical attributes, including strong absorption bands, high molar absorption coefficients, elevated quantum yields, and excellent photostability [32,33]. The absorption and emission properties of squaraine dyes can be tuned from the visible to NIR wavelength region by carefully selecting donor groups with controllable π-conjugation [34,35]. Additionally, SQ dyes exhibit a pronounced aggregation-caused quenching (AIQ) due to the presence of extended π-conjugation leading to a planer molecular framework. This allows for the modulation of their fluorescence properties by controlling their aggregation and dispersion states [36]. This characteristic has been utilized to develop a variety of fluorescent probes, including those for detecting proteins, enzymes, and ions. For instance, a BSA-SQ assembly was designed as a fluorescent “on-off” probe for pepsin detection, where the aggregation of SQ led to fluorescence quenching in the pepsin-catalyzed hydrolysis of BSA [37]. The versatility of SQ dyes in biosensing applications extends to protein detection [38], where their binding to proteins like bovine serum albumin disrupts SQ aggregation, leading to fluorescence enhancement. Furthermore, peptide-conjugated SQ probes based on Förster Resonance Energy Transfer (FRET) have been designed for enzyme detection [39], where enzymatic cleavage terminates the resonance energy transfer process and subsequently increases the fluorescence intensity. Aggregation-induced quenching (AIQ) and FRET can work synergistically to enhance biosensing systems. Hence, there is broad applicability of SQ dyes in developing sensitive and selective fluorescent probes for various biomolecules and ions, capitalizing on their unique aggregation based and energy transfer properties.

In this study, we present the development of an internally quenched homo-labeled fluorescent peptide substrate for the sensitive and selective detection of trypsin enzyme activity. The probe features two NIR-sensitive unsymmetrical squaraine dyes conjugated at each terminal of a trypsin-cleavable peptide sequence. This configuration exhibits fluorescence quenching in its intact state. Upon cleavage by trypsin, the probe demonstrates a significant enhancement of the fluorescence signal in the NIR region, enabling a sensitive detection of trypsin activity. The designed probe offers exceptional sensitivity, capable of detecting trypsin activity at exceedingly low concentrations. Moreover, it demonstrates remarkable selectivity, with minimal interference from other proteases commonly found in complex biological samples. This approach has the potential to improve diagnostics and monitoring of trypsin-related disorders and also has potential applications in proteomics research and drug discovery.

## 2. Materials and Methods

### 2.1. Reagents and Materials

All chemicals, solvents, and reagents were of analytical or spectroscopic grade and used as supplied. Fmoc-protected amino acids, Rink amide 4-methylbenzhydrylamine (MBHA)resin,piperidine,1-((Dimethylamino)(dimethyliminio)methyl)-1H-benzo[d][1,2,3]triazole 3-oxide hexafluorophosphate (HBTU), 1H-Benzo[d][1,2,3]triazol-1-ol (HOBt)·H_2_O, diisopropylethylamine (DIEA), trifluoroacetic acid (TFA), and 4 M HCl/Dioxane were purchased from Watanabe Chemical Industries, Ltd. (Hiroshima, Japan). Enzymes (α-chymotrypsin, α-trypsin from bovine pancreas, pancreatic elastase, horseradish peroxidase, bovine serum albumin, and papain) were obtained from Sigma-Aldrich Co., LLC (Tokyo, Japan). All other reagents and solvents were procured from Wako Pure Chemical Industries, Ltd. (Osaka, Japan).

### 2.2. Theoretical Structure Calculation

The theoretical quantum chemical calculation for the structural optimization of squaraine dye–peptide conjugate (**SQ-3 PC**) was conducted on a Dell workstation (8 Core) using the Gaussian G16 program package [40]. The calculation was performed for the isolated molecule in the gaseous state. In the theoretical calculations, judicious selection of a suitable basis set, theory, and functional are required to make a logical balance between the computation cost and accuracy. Considering the huge number of atoms in the molecule, theoretical calculation for structure optimization was conducted on HF/6-31G level of theory to save computation time.

### 2.3. Instrumentation

Nuclear magnetic resonance (NMR) spectroscopy (500 MHz for 1H NMR) and TOF/FAB-mass spectroscopy in positive ion monitoring mode were used for structural characterization. UV–visible–NIR absorption spectra were recorded on a JASCO V-530 UV/VIS spectrophotometer (JASCO Corporation, Tokyo, Japan). Fluorescence emission spectra were obtained using a JASCO FP-6600 spectrofluorometer (JASCO Corporation, Tokyo, Japan). Analytical HPLC was performed on a Hitachi L-7100 system (Hitachi High-Technologies Corporation, Tokyo, Japan) with an Xterra MS C18-5 μM column (4.6 × 250 mm; Waters Corporation, Milford, MA, USA). The sample was pre-incubated in a EYELA SLI-220 temperature-controlled incubator (Tokyo Rikakikai Co., Ltd., Tokyo, Japan) to ensure thermal equilibration. During spectroscopic measurements, the cuvette holder was equipped with a water-jacketed cell holder connected to a THERMO SUPPLIER EZL-80F circulating water bath system (TAITEC Corporation, Saitama, Japan) maintained at 37 °C.

### 2.4. Spectroscopic Measurements

Stock solutions of 100 μM trypsin in phosphate buffer saline (PBS) (pH 7.2, 0.1 M) and 100 μM **SQ-3 PC** in dimethyl sulfoxide (DMSO) were prepared and diluted as required. The fluorescence response of **SQ-3 PC** (5 μM) to trypsin was evaluated in H_2_O (pH 7.2, 2% DMSO). Fluorescence measurements were taken at 659 nm (excitation and emission slit widths of 5 nm and 6 nm, respectively) at 5 min and then every 10 min for 30 min. Trypsin concentrations ranged from 1 nM to 75 nM. Baseline fluorescence intensity (F_0_) was determined using 5 μM **SQ**-**3 PC** in H_2_O with 2% DMSO.

### 2.5. Determination of Detection Limit

The limit of detection (LOD) and limit of quantification (LOQ) for trypsin-specific fluorescence biosensor was calculated using the following formula:

LOD = 3.3 (SD)/S(1)

OQ = 10 (SD)/S(2)

where S is the slope of the linear part of the calibration curve and SD is the standard deviation of the sample for consecutive measurements.

### 2.6. Fluorescence Quenching Efficiency

Fluorescence quenching efficiency was calculated using the following equation:

Quenching efficiency (%) = [1 − (F_PC_/F_dye_)] × 100 (3)

where F_dye_ is the emission intensity of SQ-3 dye and F_PC_ is the emission intensity of SQ-3 PC at the same concentration in H_2_O (2% DMSO).

## 3. Results

### 3.1. Design and Synthesis of Target Molecules

**Figure 1:**
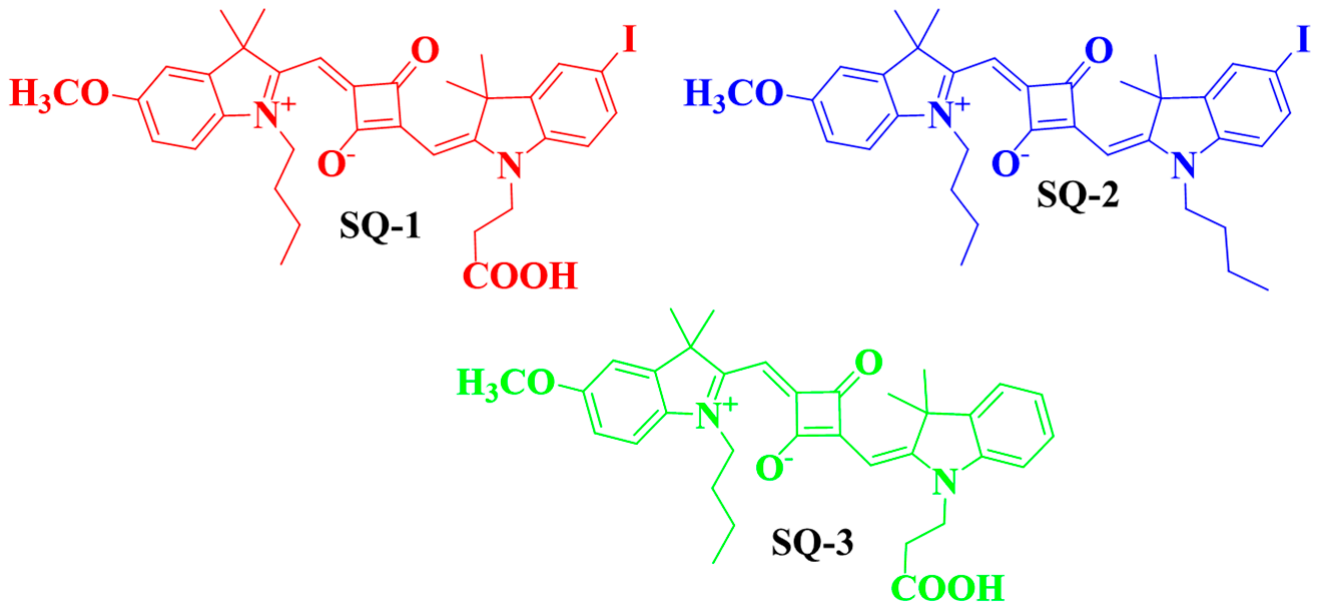
Chemical structure of squaraine dyes (**SQ-1**, **SQ-2**, and **SQ-3**).

In this study, three unsymmetrical squaraine dyes (**SQ-1**, **SQ-2**, and **SQ-3**) and a dye–peptide conjugate (**SQ-3 PC**) were synthesized and investigated as NIR-sensitive fluorescent molecules for determining trypsin enzyme activity. The dyes are based on a squaraine core with two indole-based terminal groups (Figure 1). The molecular design strategy involved several key modifications to the squaraine core structure to enhance its photophysical properties. Iodine was introduced to investigate the heavy atom effect, potentially improving intersystem crossing. A methoxy group was added as an electron-donating substituent to optimize electronic distribution within the π-conjugated system. Additionally, a propionic acid moiety was incorporated at the N-position of one terminal indole ring to establish an intramolecular charge transfer (ICT) system and provide a reactive carboxylic acid for bioconjugation with peptides via amide bond formation. **SQ-1** features an asymmetric substitution with an iodo group (-I) and a propionated alkyl chain(-CH_2_CH_2_COOH) on one indole group and a methoxy group and butyl chain(-CH_2_CH_2_CH_2_CH_3_) on the other. **SQ-2** is similar to **SQ-1** but has butyl chains on both indole groups. **SQ-3** resembles **SQ-1** but lacks the iodine substitution. The synthesis, structural characterization, and analytical data for these compounds and their intermediates are provided in the Supplementary Materials (Schemes S1 and S2).

**Figure 2:**
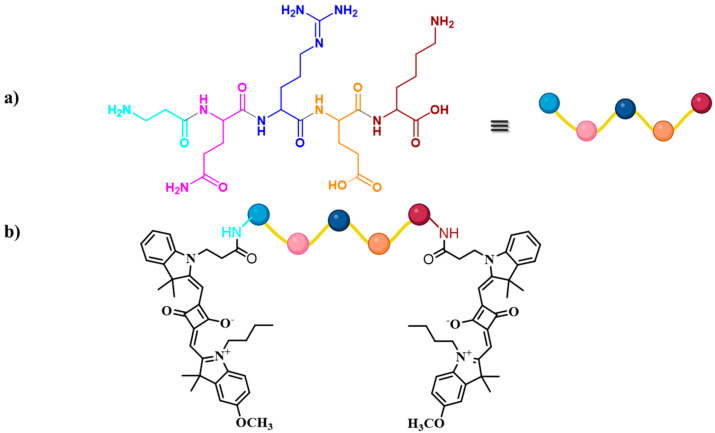
Chemical structure of (**a**) trypsin enzyme-specific peptide and (**b**) dye–peptide conjugate probe (**SQ-3 PC**) for the trypsin enzyme sensing.

**SQ-3** was identified as the optimal choice for the preparation of the dye–peptide conjugates due to its distinctive structural attributes, aggregation properties, and optical behavior. Although the substitution of hydrogen with a heavy atom such as iodine in squaraine dyes has been previously reported to enhance fluorescence quantum yield [41], this modification did not prove to be significantly advantageous in the present study. The absence of iodine in **SQ-3** avoids potential heavy metal effects and reduces steric hindrance, potentially facilitating a more efficient coupling to peptides and better interaction with the target enzyme trypsin. In a recent study (Soumya et al.) [42], a symmetrical iodinated squaraine dye is designed to be a photosensitizer for photodynamic therapy, where its stability is crucial for targeted action on tumor tissues. This stability may contribute to its resistance to enzyme hydrolysis. **SQ-3** exhibits the highest aggregation index (AI) in PB of 3.38, attributed to the combination of electron-donating (-OCH_3_) and electron-withdrawing (-COOH) groups promoting intermolecular interactions. The amino acid sequence Gln-Arg-Glu was chosen as the substrate for the trypsin enzyme based on the known specificity of trypsin [43]. Specifically, trypsin cleaves peptide bonds between the carboxyl group of arginine or lysine and the amino group of the adjacent amino acid. To optimize the design of our biosensing probe, we incorporated additional structural elements (Figure 2a). Free amino groups with β-Ala and Lys were integrated to serve dual purposes: first, as spacers to provide flexibility towards the substrate, and second, to facilitate the binding of the **SQ-3** squaraine dye to the substrate.

**Scheme 1:**
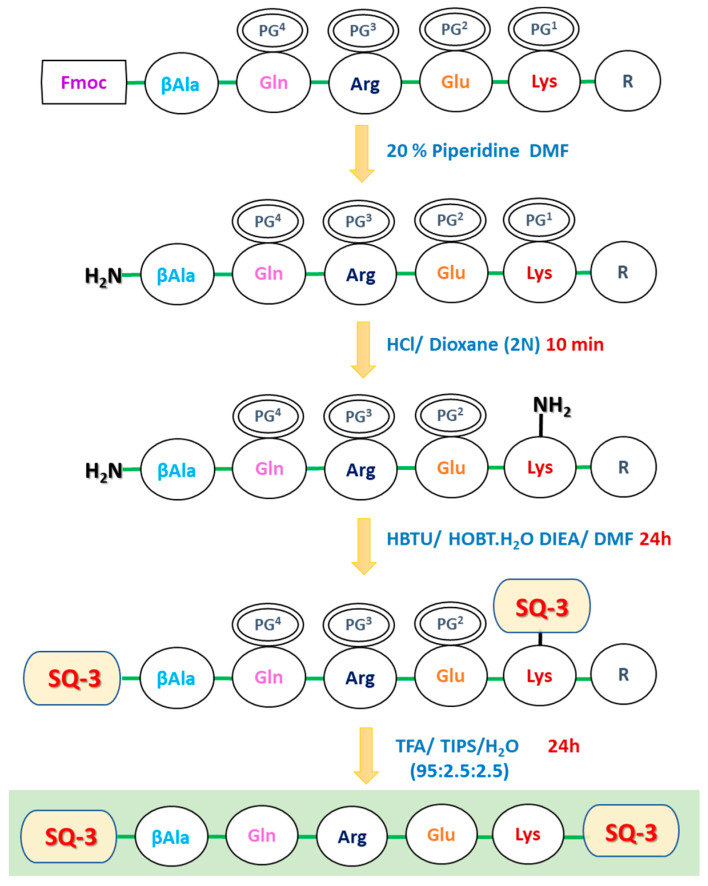
Schematic representation for the synthesis of dye–peptide conjugate (**SQ-3 PC**); R, Rink Amide MBHA Resin; PG^1^, Boc; PG^2^, o’tBu; PG^3^, Pbf; PG^4^, trt.

The trypsin-specific probe **SQ-3-PC** (Figure 2b) was synthesized using solid-phase peptide synthesis (SPPS). The design incorporated a linear Gln-Arg-Glu tripeptide, chosen for trypsin specificity, with β-Alanine as a spacer and Lysine for the side-chain binding of the terminal squaraine dye. Synthesis began with Fmoc-Lys(Boc)-OH conjugation to Rink Amide MBHA Resin, followed by a stepwise peptide chain construction (Scheme 1).The resin-supported peptide (Fmoc-βAla-Gln(trt)-Arg(Pbf)-Glu(o′tBu)-Lys(Boc)-Resin) underwent Fmoc deprotection with 20% piperidine and Boc removal with HCl/Dioxane (2 M). **SQ-3** was then conjugated at a 2.2:1 molar ratio using HOBt/HBTU as coupling reagents. The final cleavage and deprotection were achieved using a TFA/triisopropylsilane/H_2_O (95:2.5:2.5) cocktail. The crude dye–peptide conjugate was precipitated with ether and purified by silica gel column chromatography (chloroform/methanol, 9:1), yielding the final probe. Successful synthesis was confirmed by Time-of-Flight (TOF) mass spectrometric analysis, which revealed a measured *m*/*z* of 1702.9112 [M + H]^+^ (calculated 1701.8901). The detailed synthesis procedure, reaction conditions, and analytical data, including mass spectrometry, are provided in the Supporting Information (Scheme S3).

### 3.2. Optical Characterization of Squaraine Dyes and Dye–Peptide Conjugate

**Figure 3:**
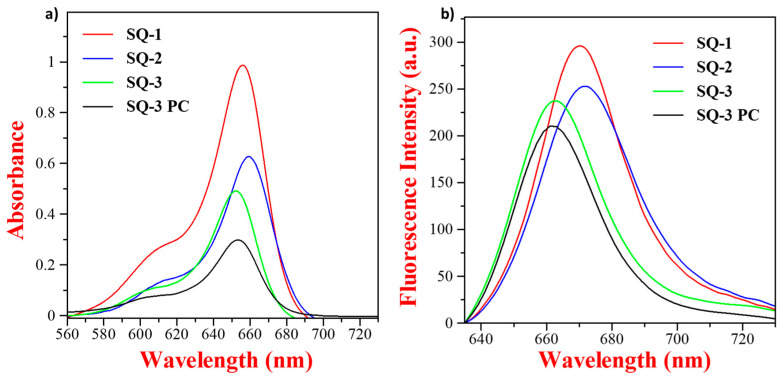
(**a**) Optical absorption and (**b**) fluorescence emission spectra of **SQ-1**, **SQ-2**, **SQ-3**, and **SQ-3 PC** in DMSO (5 μM).

The photophysical properties of squaraine dyes (**SQ-1**, **SQ-2**, **SQ-3**, and **SQ-3 PC**) were characterized using optical absorption and fluorescence emission spectroscopies in DMSO. These measurements aimed to elucidate the impact of structural modifications on the optical properties. Figure 3a,b present the absorption and emission spectra, respectively, at a dye concentration of 5 µM in DMSO along with the summarization of the deduced optical parameters such as absorption maximum (λ_abs_), emission maximum (λ_em_), molar extinction coefficients (ε), and Stokes shifts (Δ) in Table 1. All dyes exhibit strong and sharp optical absorption with the absorption maximum (λ_abs_) between 652 nm and 659 nm with a weak vibronic shoulder between 600 nm and 620 nm, characteristic of squaraine dyes due to the π-π* transition. **SQ-3**, with -OCH_3_ and -COOH groups, has a strong, narrow band in the visible to NIR wavelength region. **SQ-1** and **SQ-2**, containing iodine and -OCH_3_ groups on the main ring, show relatively higher bathochromic shifts due to the heavy atom effect, broadening the absorption band and shifting it to longer wavelengths, which is a similar effect as observed by Mayerhöffer et al. [41].

**Figure 4:**
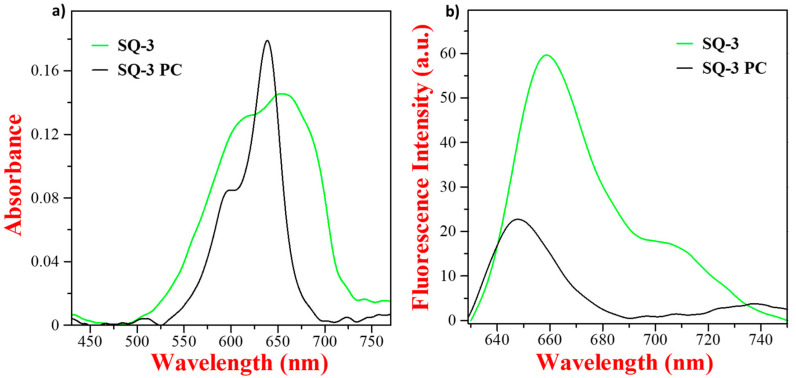
(**a**) Absorption and (**b**) emission Spectra of **SQ-3** and **SQ-3 PC** (5 μM) in H_2_O (2% DMSO) solvent.

These structural differences not only affect the optical absorption characteristics but also influence the emission properties of the dyes under investigation, as shown in Figure 3b. The enhanced emission of iodine-containing **SQ-1** and **SQ-2** is likely due to the iodine heavy atom effect, which may increase the molecular rigidity or reduce non-radiative decay. **SQ-1** with a single iodine atom achieves an optimal balance between the heavy atom effect and structural asymmetry, resulting in the highest emission intensity. The absence of iodine in **SQ-3** may lead to greater molecular flexibility, increased non-radiative decay, and lower emission intensity. Nevertheless, all of the dyes depict a very small Stokes shift of only 8 nm–15 nm with similar shapes of absorption and emission spectra, which are indicative of the very comparable molecular configurations of the dye in the ground and excited states [44]. Interestingly, the optical behavior observed in the aqueous medium is very different compared to that observed in the DMSO solution. The electronic absorption spectrum of **SQ-3** in H_2_O containing 2% DMSO showed a broad absorption spectral feature with **λ_abs_** at 653 nm and a highly pronounced vibronic shoulder appearing at 610 nm, as shown in Figure 4a, indicating an enhanced dye aggregation in the aqueous medium.

**Figure 5:**
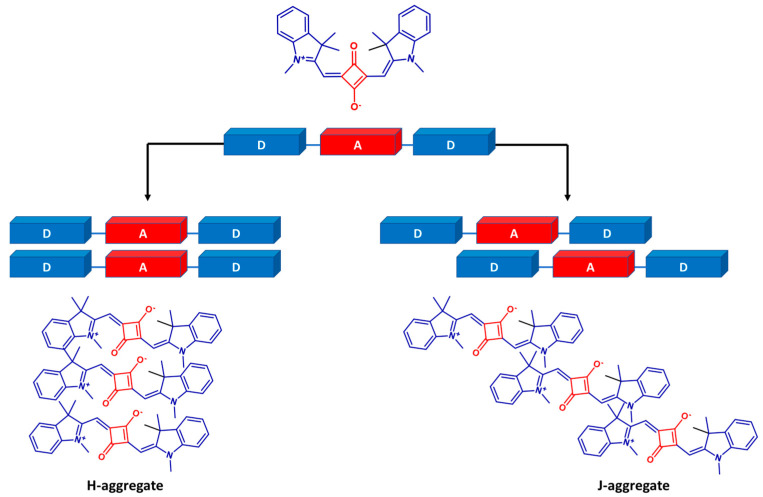
Schematic representation of H- and J-aggregate formation in squaraine dyes.

In contrast, **SQ-3 PC** in the same aqueous medium exhibits a blue-shifted, sharp absorption peak at 639 nm, with a prominent vibronic shoulder at 598 nm and less spectral broadening compared to **SQ-3**. This relatively lesser extent of molecular aggregation in **SQ-3 PC** compared to that of **SQ-3** is attributed to the hampering of aggregation due to the presence of the oligopeptide chain and spacers. Squaraine dyes are prone to dye aggregation forming blue-shifted and co-facial H-aggregates and red-shifted head-to-tail packed J-aggregates (Figure 5) compared to monomeric dye absorption, which is influenced by the structure and molecular environment of the dyes’ planar molecular structure [45]. The vibronic shoulder in squaraine dyes is very sensitive to dye aggregation and quite often has been used to index the relative extent of dye aggregation in different molecular environments. In the electronic absorption spectrum of squaraine dyes, the ratio of the absorbance around 600 nm associated with vibronic shoulder and around 650 nm associated with the monomeric dye absorption has been widely utilized to estimate the relative extent of the dye aggregation [46]. A perusal of the normalized absorption spectra of **SQ-3** and **SQ-3 PC** in DMSO and water (Supporting Information Figure S1) reveals that the extent of aggregation of both of the dyes in DMSO is almost similar (0.26), while the behavior is very different in water, with nearly a doubled aggregation index for **SQ-3** dye (0.91) compared to that of **SQ-3 PC** (0.46). At the same time, the nature of the aggregates is primarily H-type [45] in **SQ-3 PC**, while **SQ-3** demonstrated the presence of both the H- and J- aggregates [47]. The unexpectedly low molar absorption coefficient of **SQ-3 PC**, despite containing two squaraine moieties, can be attributed to intramolecular electronic coupling leading to hypochromism, non-optimal conformations due to the flexible linker, and specific solute–solvent interactions in DMSO. These factors collectively contribute to a reduced absorption intensity compared to the expectations for two independent squaraine chromophores.

**Figure 6:**
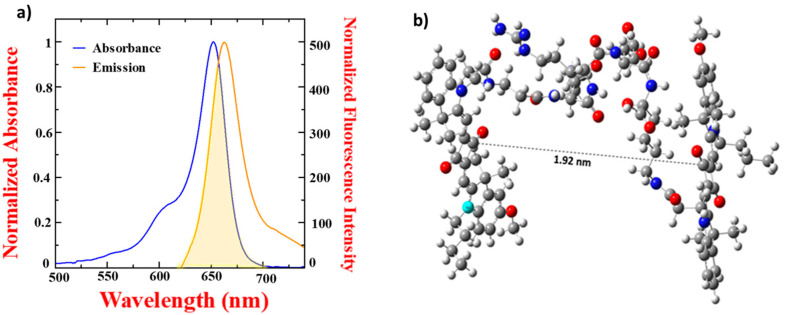
(**a**) Normalized absorption and emission spectra of **SQ-3** in DMSO and (**b**) optimized molecular structure of **SQ-3 PC** calculated using Gaussian G16 program.

Notably, in the aqueous solution, **SQ-3-PC** and **SQ-3** exhibited a quenched fluorescence nearly ten times and five times higher (Figure 4b), respectively, than that observed in the DMSO solution. The quantum yield of **SQ-3** and **SQ-3 PC** in water was calculated and found to be 0.006 and 0.0026. This is associated with aggregation-induced quenching (AIQ) due to the enhanced intermolecular interaction in water facilitated by hydrogen bonding between the free -COOH of the dye and water molecules. It is interesting to note that despite the nearly halved aggregation in **SQ-3 PC** in water compared to that of pure dye **SQ-3**, the quenching in fluorescence for **SQ-3 PC** was about three times higher compared to that of **SQ-3**. This clearly corroborates that, apart from AIQ, there might be an alternative pathway for fluorescence quenching in the case of **SQ-3 PC**. A second possibility is the quenching caused by fluorescence resonance energy transfer (FRET). To have FRET-based quenching, two criteria, such as the presence of the two terminal fluorophores within the Forster radius of typically 2 nm–20 nm and a sufficient overlap between the absorption spectrum of one fluorophore with the emission spectrum of another one, need to be fulfilled [41]. Due to a very small Stokes shift of only 11 nm in the case of **SQ-3** dye in DMSO, there is a sufficient overlap between the absorption and emission spectrum of **SQ-3**, as shown in Figure 6a fulfilling the first criterion of FRET in the case of **SQ-3 PC**. Interestingly, the distance between the two faces of **SQ-3** dye in the dye–peptide conjugate **SQ-3 PC** is only 1.92 nm (Figure 6b), which is well within the typical Förster radius, ensuring the efficient energy transfer from one dye molecule to another, fulfilling the second criterion for FRET to occur. By analyzing the fluorescence intensities of the dye (**SQ-3**) and the dye–peptide conjugate (**SQ-3 PC**), we determined that the fluorescence quenching efficiency was 61.8% in H_2_O (2% DMSO). Therefore, comparing the AIQ and FRET mechanisms present in **SQ-3 PC**, FRET seems to be more dominant compared to AIQ. The property of conjugation allowed us to investigate the potential application of dye–peptide conjugates for enzyme activity monitoring using fluorescence-based ON/OFF biosensing.

### 3.3. Enzymatic Hydrolysis of SQ-3-PC with Trypsin

**Figure 7:**
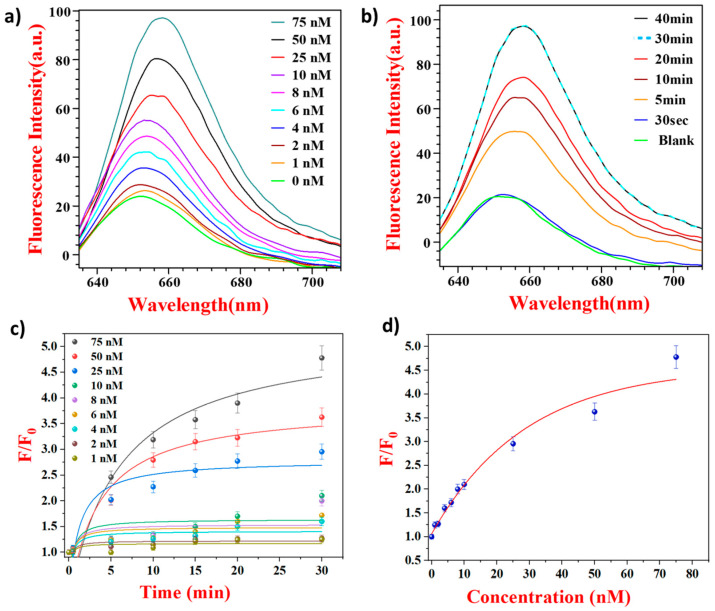
(**a**) Concentration-dependent fluorescence spectra of **SQ-3 PC** (5 µM) in H_2_O (2% DMSO) for 30 min with the addition of 0 to 75 nM Trypsin, (**b**) time-dependent fluorescence spectra of **SQ-3PC** (5 µM) in H_2_O (2% DMSO) for 30 min with the addition of 75 nM of trypsin enzyme, (**c**) fluorescence spectra of **SQ-3 PC** with change in fluorescence intensity as a function of time with different concentrations of enzyme for a fixed concentration of **SQ-3 PC** (5 μM), and (**d**) plot of ratio of fluorescence intensity (F/F_0_) as a function of enzyme concentration.

The newly designed NIR-sensitive probe **SQ-3-PC** exhibited a highly quenched fluorescence signal caused by the AIQ and FRET, which later played a dominant role. However, after incubation with the trypsin enzyme, there was a substantial increase in fluorescence at 654 nm associated with monomeric absorption. Upon the addition of varying concentrations of this enzyme prepared in the phosphate buffer with a (pH of 7.4, 0.1 mM) in a 5 μM solution of **SQ-3-PC** in H_2_O (2% DMSO) followed by incubation for 30 min, there was a significant restoration of quenched fluorescence of the dye, as shown in Figure 7a. A perusal of Figure 7b shows that there was a significant increase in the fluorescence of the dye within 5 min. After 30 min, the fluorescence reached saturation, resulting in a 5-fold increase compared to the fluorescence in the absence of the trypsin enzyme. The observed enhancement in fluorescence intensity subsequent to enzymatic hydrolysis can be attributed to the spatial separation of the **SQ-3** fluorophores beyond their Förster radius. This increased intermolecular distance effectively terminates the resonance energy transfer process, resulting in the cessation of FRET and the concomitant restoration of fluorescence emission. Figure 7c,d show that F_0_ indicates the initial fluorescence intensity in the absence of the enzyme, where F represents the fluorescence intensity at a specified point of time after the enzyme is added. Figure 7c demonstrates the fluorescence response of the squaraine-based probe **SQ-3 PC** (5 μM) to varying concentrations of the target enzyme (0–75 nM). The fluorescence intensity changes as a function of enzyme concentration, with higher enzyme levels inducing more rapid and pronounced spectral shifts. This indicates a direct correlation between the enzyme concentration and probe activation. Concentration-dependent changes in the fluorescence (F/F_o_) at the emission maximum, as shown in Figure 7d, reveal that **SQ-3 PC** works as a trypsin enzyme-specific NIR fluorogenic probe with the LOD and LOQ of 1.07 nM and 3.25 nM, respectively. The linear regression equation was y = 0.11327x + 1.05815, R^2^ = 0.97043 (Figure S2). Our innovative approach for trypsin detection demonstrates significant advancements over existing methodologies. The developed probe exhibits a remarkably low LOD, which is comparable to or surpasses the most sensitive techniques reported in the recent literature (Table S1). In healthy humans, the concentration of trypsin varies in serum and in the intestine. In serum under fasting conditions, the concentration of trypsin varies from 4 nm to 30 nM [48,49]. Pancreatic diseases such as cystic fibrosis, acute pancreatitis, or the acute phase of chronic pancreatitis are associated with an increased trypsin level of 2.1–71.42 nM in the serum of patients [50]. Thus, the synthesized probe is able to measure the trypsin level in healthy individuals and individuals with a disease associated with the hyper- and hypo-activities of the trypsin enzyme. Furthermore, the detection of trypsin in the NIR wavelength regions allows the utilization of direct body fluids without prior sample processing, owing to the highly diminished autofluorescence.

### 3.4. Enzyme Selectivity of the Enzyme Probe SQ-3-PC

**Figure 8:**
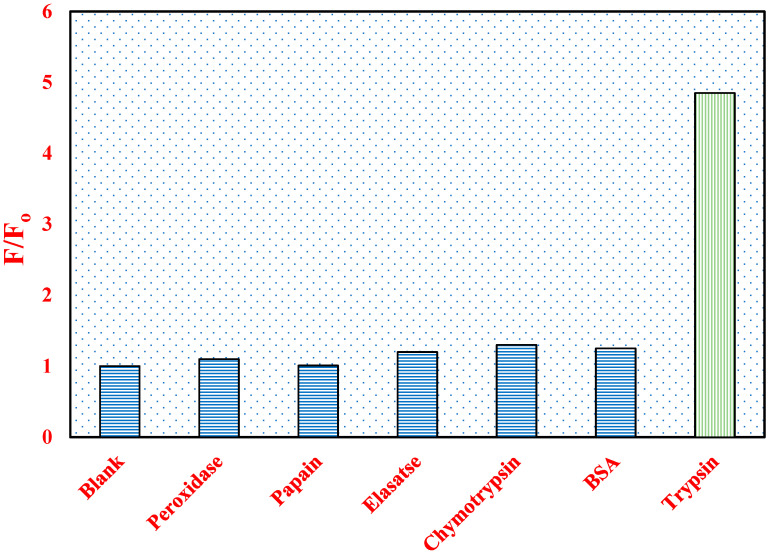
Fluorescence response (F/F_0_) of the probe **SQ-3 PC** (5 μM) in H_2_O (2% DMSO) towards different potential and competing enzymes (75 nM).

Apart from the sensitivity and detection limit, the selectivity of a probe is highly desired for targeted disease diagnosis and point-of-care testing devices. The selectivity of the present probe towards trypsin was evaluated by analyzing its response in the presence of potentially interfering enzymes. A 5 µM solution of the newly designed probe **SQ-3 PC** in H_2_O (2% DMSO) was incubated with various enzymes, including trypsin, papain, pancreatic elastase, horseradish peroxidase, chymotrypsin, and bovine serum albumin (BSA), each at a concentration of 75 nM, for 30 min. As shown in Figure 8, the emission intensity at 654 nm remained relatively constant in the presence of the competing enzymes. However, a significant increase in emission intensity was observed upon the addition of trypsin. This marked change in fluorescence signal highlights the probe’s high selectivity for trypsin, demonstrating its ability to specifically detect trypsin activity while remaining unresponsive to other enzymes present in the sample. These results underscore the potential of this biosensing approach for accurate trypsin detection in complex biological environments.

## 4. Conclusions

In conclusion, we have successfully designed and synthesized novel unsymmetrical squaraine dyes and a potential dye–peptide conjugate for the sensitive and selective detection of the trypsin enzyme. In the aqueous medium, the dye–peptide conjugate **SQ-3 PC** demonstrates reduced dye aggregation but pronounced fluorescence quenching compared to its constituent dye **SQ-3** alone. A fluorescence quenching of 61.8% in the case of **SQ-3 PC** in an aqueous medium compared to its DMSO counterpart is associated with both the AIQ and FRET, where FRET was demonstrated to be the dominant phenomenon for this pronounced fluorescence quenching. The hydrolysis of the designed probe **SQ-3 PC** with trypsin enzyme restores an appreciable amount of the quenched fluorescence within 5 min, and the enhancement in the fluorescence saturates after 30 min. Notably, the probe exhibited high specificity and good selectivity towards trypsin when tested against a panel of potentially interfering enzymes, including BSA, papain, chymotrypsin, horseradish peroxidase, and pancreatic elastase. This selectivity is crucial for the potential application of the probe in complex biological samples, though further validation in such matrices remains an important area for future investigation.

## Figures and Tables

**Table 1:** Optical parameters for the squaraine dyes and dye–peptide conjugates in DMSO solution.

Squaraine Dye	λ_abs_	λ_em_	Δ	ԑ (M^−1^cm^−1^)
**SQ-1**	656 nm	671 nm	15	1.98 × 10^5^
**SQ-2**	659 nm	672 nm	13	1.26 × 10^5^
**SQ-3**	652 nm	663 nm	11	1.00 × 10^5^
**SQ-3 PC**	654 nm	662 nm	8	0.60 × 10^5^

## Data Availability

The data presented in this study are available on request from the corresponding author. The data are not publicly available due to ethical and privacy reasons.

## Supplementary Materials

The following supporting information can be downloaded at: https://www.mdpi.com/article/10.3390/bios14100458/s1, Scheme S1: Scheme for synthesis for unsymmetrical squaraine dye **SQ-1**, **SQ-2**; Scheme S2: Scheme for synthesis for unsymmetrical squaraine dye **SQ-3**; Scheme S3. Scheme for the synthesis of peptide sequence; Figure S1: Normalized Absorption Spectra of **SQ-3** and **SQ-3** PC in DMSO (solid line) and H_2_O (2% DMSO) (dash line) at a concentration of 5 µM.; Figure S2: Linear correction curve of ratio of fluorescence intensity against trypsin concentration (0 nM to 10 nM). Table S1: Comparison of different methods for the determination of trypsin.
