# Supplementary material for: NIR-Sensitive Squaraine Dye—Peptide Conjugate for Trypsin Fluorogenic Detection
Source: Biosensors (Basel). 2024 Sep 25;14(10):458. doi: 10.3390/bios14100458 (PMC11505658; doi:10.3390/bios14100458)
Supplement: Supplementary file 1 [file biosensors-14-00458-s001.zip › biosensors-3163075-supplementary.pdf]

NIR-Sensitive Squaraine Dye–Peptide Conjugate for Trypsin Fluorogenic Detection

Priyanka Balyan, Shekhar Gupta, Sai Kiran Mavileti, Shyam S. Pandey * and and Tamaki Kato *

Graduate School of Life Science and System Engineering, Kyushu Institute of Technology,2-4 Hibikino,
Wakamatsu-Ku, Kitakyushu-Shi, Fukuoka, 808-0196, Japan
* Correspondence: shyam@life.kyutech.ac.jp; tmkato@life.kyutech.ac.jp

Experimental

S1. Synthesis of Unsymmetrical Squaraine dye SQ-1, SQ-2.

Unsymmetrical squaraine dyes SQ-1, SQ-2 along with their respective intermediates were synthesized as per the synthetic scheme shown in Scheme S1.

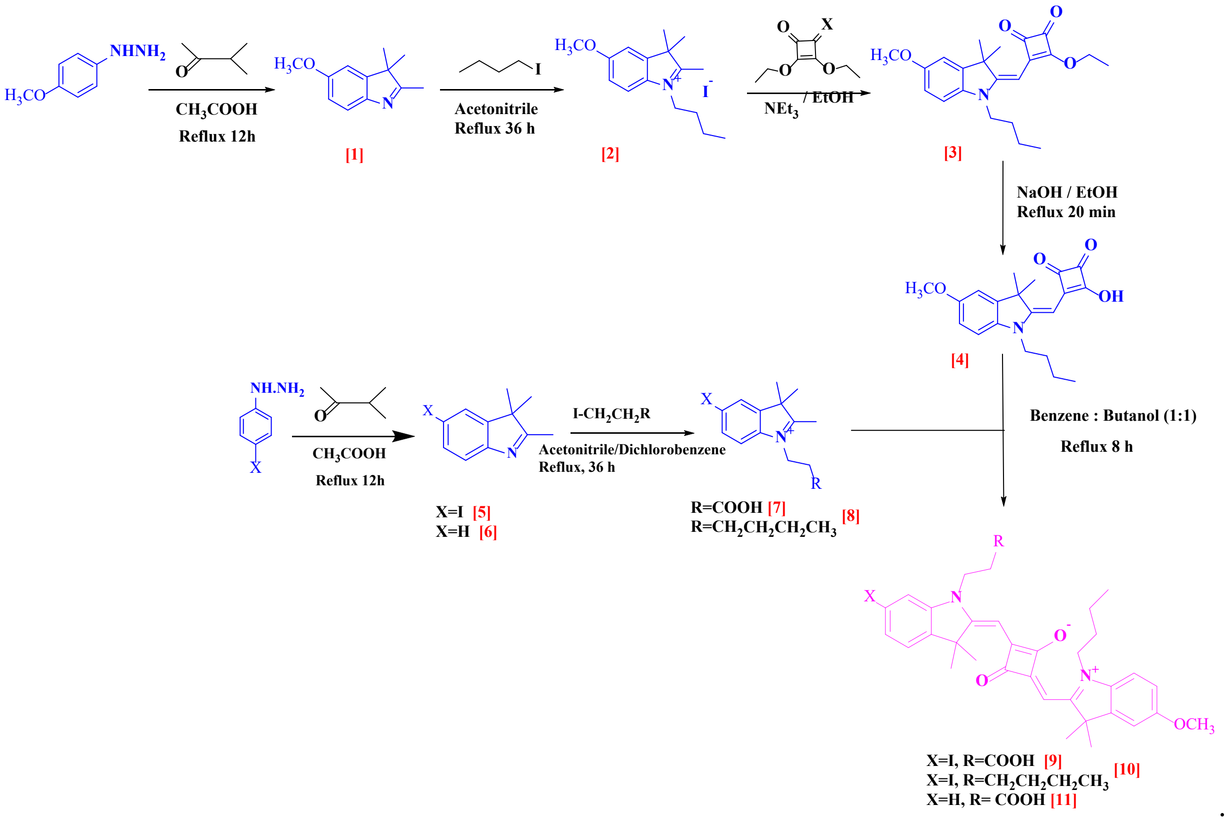

Scheme S1. *Scheme for synthesis for unsymmetrical squaraine dye* **SQ-1, SQ-2.**

S2. Synthesis of Unsymmetrical Squaraine dye SQ-3

Unsymmetrical squaraine dye SQ-3 along with their respective intermediates were synthesized as per the synthetic scheme shown in Scheme S2.

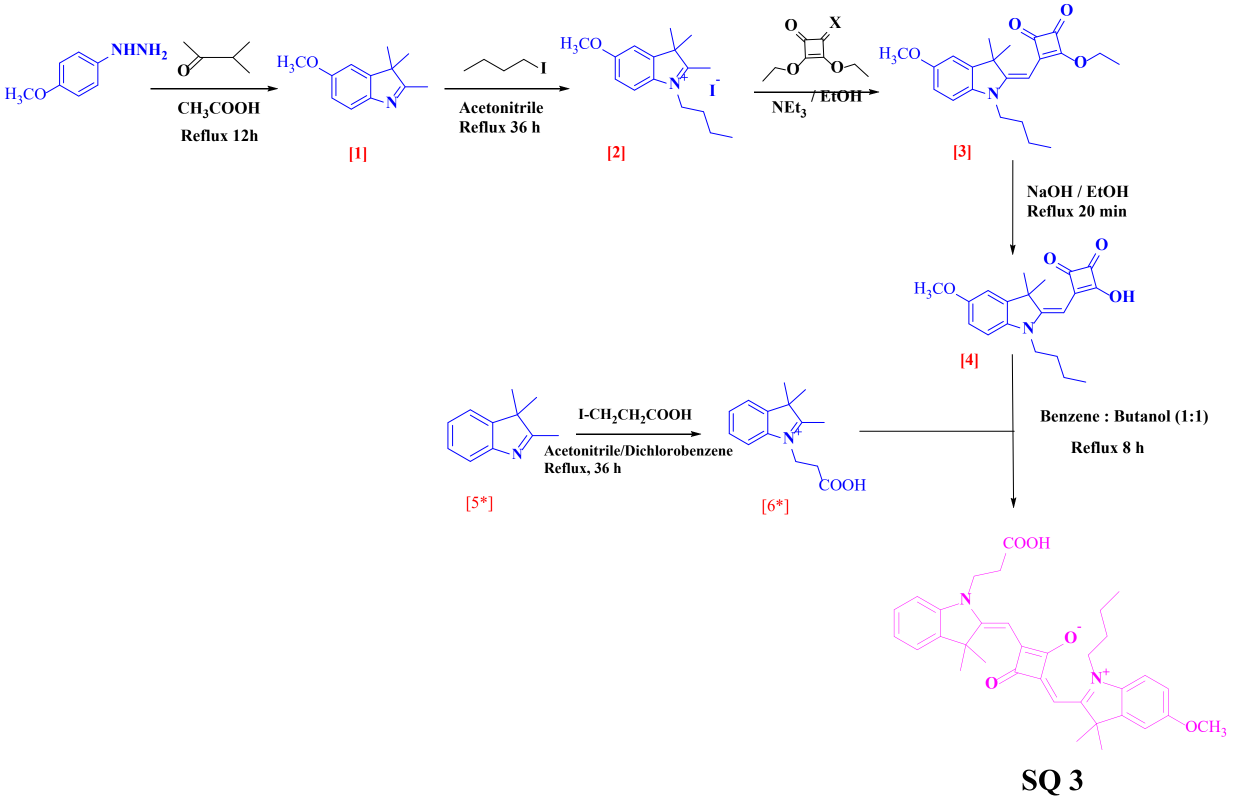

Scheme S2. *Scheme for synthesis for unsymmetrical squaraine dye SQ-3.*

S1. Synthesis of Squaraine Dyes (SQ-1, SQ-2, SQ-3)

S1.1 Synthesis Of 5-Methoxy-2,3,3-Trimethyl 3H–Indolium [1] and1-Butyl-5-Methoxy-2,3,3-Trimethyl-3H-Indolium [2]

3-methyl-2-butanone (10.9 g, 125 mmol) was added to a round-bottom flask containing 50 mmol of 4-methoxy phenyl hydrazine hydrochloride (8.6 g) in 80 mL of glacial acetic acid. The mixture was refluxed for 18 hours. The solvent was evaporated under reduced pressure and the concentrated compound was extracted with chloroform, leading to the targeted compound as a brown viscous liquid with 94% yield. To the compound[1], 2,3,3-trimethyl-3H-indole-5-methoxy (1 equiv.) 1-iodobutane (3 equiv.) was dissolved in acetonitrile, and the reaction was refluxed for 36 h at 90^{0}C. The reaction was monitored by TLC using a chloroform/methanol (9:1) system. Upon

the completion of the reaction, the solvent was evaporated under reduced pressure and the product was precipitated with an ample addition of ether. The solid was filtered and dried, yielding 81%. TOF-MS (measured 246.185 [M]+; calculated m/z: 246.37) confirms the synthesis of this intermediate.

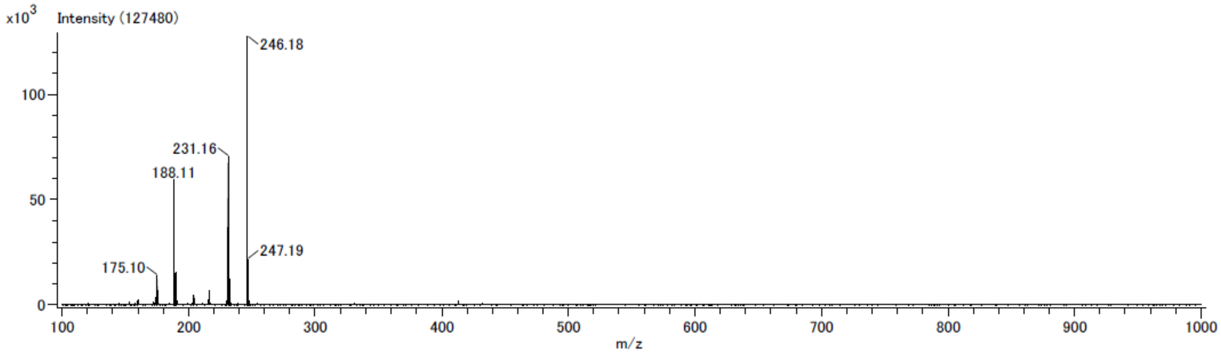

TOF-Mass of ***Compound-2***

S1.2. Synthesis of 5-Methoxy-2,3,3-Trimethyl-1-Butyl Semisquaraine Ethyl-Ester [3] and Its Hydrolyzed Product [4]

Compound **[2]** (10 mmol), 3,4-diethoxy-3-cyclobutene-1,2-dione (20 mmol, 3 mL), triethylamine (1 mL), and ethanol (50 mL) were taken in a round-bottom flask fitted with a condenser. The reaction mixture was refluxed for 8 hours, with the progress monitored by TLC. Upon completion (quenching), the reaction yielded a greenish solution. The mixture was concentrated using a rotary evaporator, and the crude product was purified by flash column chromatography using ethyl acetate/hexane as the eluting solvent, resulting in compound **[3]** as a yellow solid with a 50% yield.

This semisquaraine ester **[3]** was then hydrolyzed using 40% NaOH in ethanol for 30 minutes. After solvent removal, HCl was added for neutralization. The product was extracted with ethyl acetate and washed thoroughly with 10% Na2CO3 aqueous solution. The extract was concentrated to yield an orange solid. TOF-MS measurements of 342.185 confirmed the identity of the synthesized product.

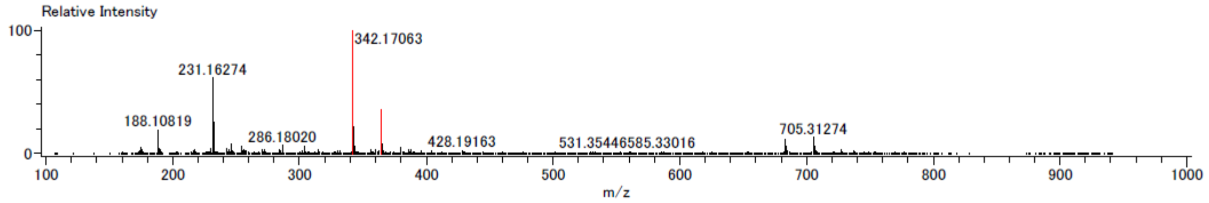

TOF- Mass of ***Compound-4***

S1.3. Synthesis of 2,3,3-Trimethyl-5-Iodo-3H-Indole [5] and 5-Iodo-1-(2-Carboxyethyl)-2,3,3-Trimethyl-3H-Indolium [6]

3-Methyl-2-butanone (10.9 g, 125 mmol) was added to a round-bottom flask containing 50 mmol of 4-iodophenyl hydrazine (8.6 g) in 80 mL of glacial acetic acid. The mixture was refluxed for 12 hours. After completion of the reaction, the mixture was cooled down and neutralized with 1 M Na_2CO_3. The solution was then diluted with H_2O, and the compound was extracted with chloroform. The chloroform was evaporated to yield the target compound as a brown viscous liquid with an 86% yield.

In a round-bottom flask (RBF) fitted with a condenser, compound **[5]** (6 g, 21 mmol) and 1-iodopropionic acid (5.199 g, 26 mmol) were dissolved in 1,2-dichlorobenzene (30 mL). The reaction mixture was heated at 140°C for 18 hours to give the corresponding 5-iodo-1-(2-carboxyethyl)-2,3,3-trimethyl-3H-indol-1-ium. After the completion of the reaction, the crude product was reprecipitated and washed with an ample amount of ether, yielding the titled compound. The product's identity was confirmed by TOF-MS (m/z: calculated 358.029).

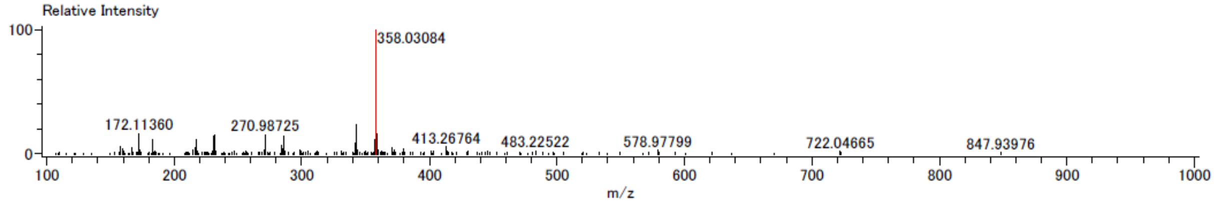

TOF- Mass of ***Compound-6***

S1.4. Synthesis of 1-Butyl-5-Iodo-2,3,3-Trimethyl-3H-Indolium [7]

2,3,3-Trimethyl-3H-indole-5-Iodo (1.0 equiv.) and 1-iodobutane (3.0 equiv.) were dissolved in acetonitrile (20 mL per gram of indole derivative). The reaction mixture was refluxed at 90°C for 36 h under an inert atmosphere. The reaction progress was monitored via thin-layer chromatography (TLC) using a chloroform/methanol (9:1 v/v) mobile phase. Upon completion, the solvent was removed in vacuo, and the crude product was precipitated by the addition of anhydrous diethyl ether (50 mL per gram of crude product). The precipitate was isolated via vacuum filtration, washed with cold diethyl ether (3 × 10 mL), and dried under high vacuum to yield the product as a crystalline solid (81% yield). The identity of the intermediate was confirmed by (TOF-MS): m/z 342.07.

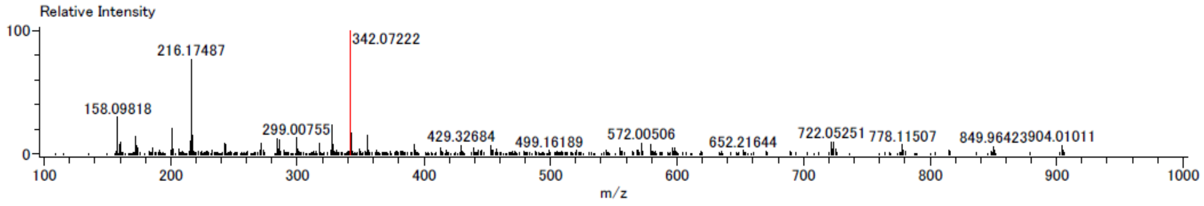

TOF-Mass of ***Compound-7***

S1.5. Synthesis (2-Carboxyethyl)-2,3,3-Trimethyl-3H-Indolium [8]

In a round-bottom flask (RBF) equipped with a condenser, compound **[1]** (6.0 g, 21 mmol) and 1-iodopropionic acid (5.199 g, 26 mmol) were dissolved in 1,2-dichlorobenzene (30 mL). The reaction mixture was heated at 140°C for 18 h under an inert atmosphere. Upon completion, the reaction yielded 5-iodo-1-(2-carboxyethyl)-2,3,3-trimethyl-3H-indol-1-ium. The crude product was isolated via reprecipitation and washed thoroughly with an ample amount of diethyl ether. The identity of the compound was confirmed by high-resolution time-of-flight mass spectrometry (TOF-MS): m/z calculated for [M]+ 231.1331, observed [M+1]+ 232.15.

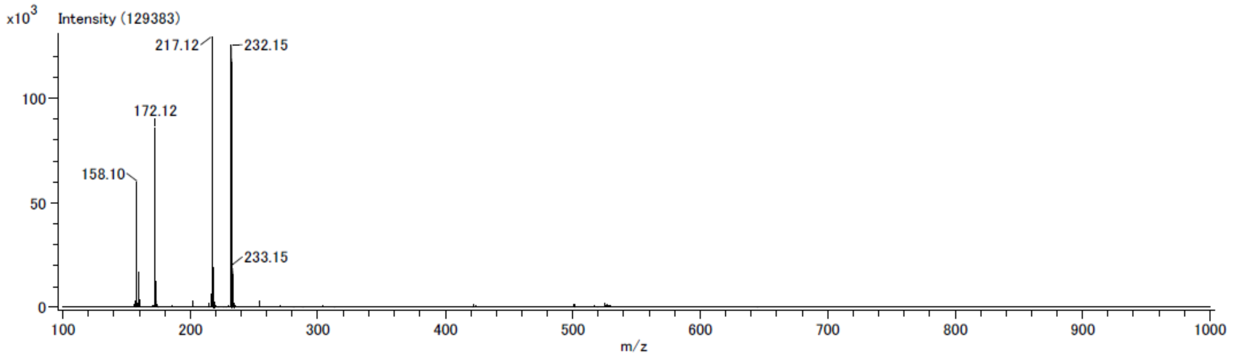

*TOF-MS of **Compound-8***

S1.6. Synthesis of Unsymmetrical Dye SQ-1 [9]

A total of 2.93 mmol (1.4g) of the intermediate (6) and hydrolyzed semi-squaraine dye intermediate (4) were dissolved in 30 mL toluene/butanol (1:1) in a round-bottom flask fitted with a condenser. The reaction mixture was then refluxed for 8 hours followed by solvent removal with the aid of a rotary evaporator. Crude dye was then subjected to flash column chromatography using CHCl3/MeOH (9:1) as the eluting solvent, giving titled dye SQ-1 as a blue solid in 86% yield. TOF-MS (measured 681.18 [M+H]+; 680.17 calculated). 1H NMR (500 MHz, CDCl3): 0.93-0.90 (t, CH_3) ; 1.28 (s, CH_3) ; 1.40-1.34 (m, CH_2) ; 1.70-1.69 (s,CH_3); 2.67-2.648 (t, CH_2) ; 2.77-2.74 (t, CH_2 - N); 3.78 (s, O-CH_3); 4.25-4.22 (t, CH_2) ; 5.88 (s, CH methylene); 6.06 (s, CH methylene); 6.80-6.78 (dd, CH); 6.79-6.78 (d, CH benzene); 6.87-6.86 (d, CH); 6.91-6.81 (d, CH benzene); 7.19 (s, CH benzene); 7.52 (s, CH benzene) confirms the identity of the synthesized product.

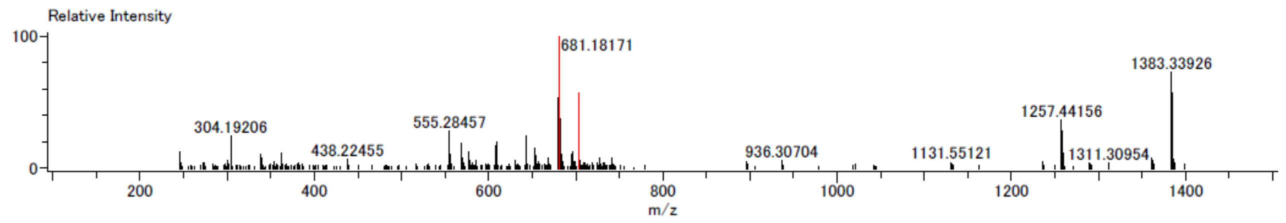

*TOF-Mass of **SQ-1***

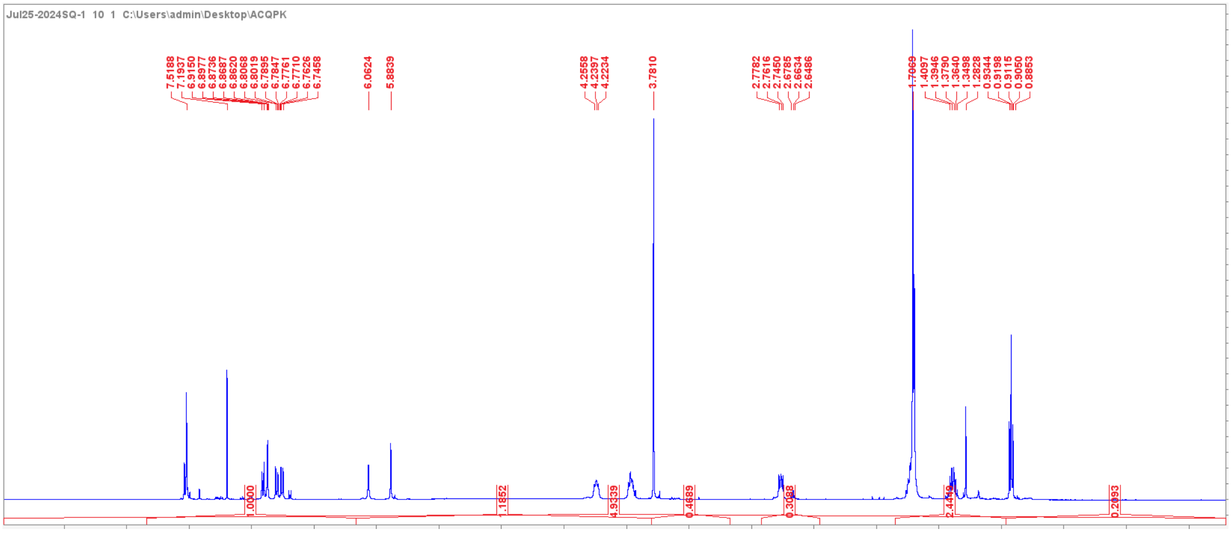

1H NMR of ***SQ-1***

S1.7. Synthesis of Unsymmetrical Dye SQ-2 [10]

A total of 1.7 mmol (800mg) of the intermediate (7) and hydrolyzed semi-squaraine dye intermediate (4) were dissolved in 30 mL toluene/butanol (1:1) in a round-bottom flask fitted with a condenser. Reaction mixture was then refluxed for 8 hours followed by solvent removal with the aid of rotary evaporator. The crude dye was then subjected to flash column chromatography using CHCl3/MeOH (9:1) as the eluting solvent, giving titled dye SQ-2 as a blue solid in 82% yield. TOF-Mass (measured 665.22 [M+H] +; 664.21 calculated). 1H NMR (500 MHz,CDCl3): δ/ppm = 0.89-0.93 (t, CH_3) ; 1.18 (s, CH_3) ; 1.35 (s, CH_3) ;1.37-1.42 (m,CH_2);1.71(s,CH3);3.75(s,O-CH_3)3.77-3.78(t,CH_2-N);3.91-3.96(t,CH2-N);5.43(s,CH ethylene);5.74(s,CH methylene) 7.19(s, CH benzene)7.43(d, CH benzene); 7.45(d, CH benzene); 7.47 (d, CH benzene); 7.49(d, CH benzene), 7.50(s, CH benzene) confirms the identity of the synthesized product.

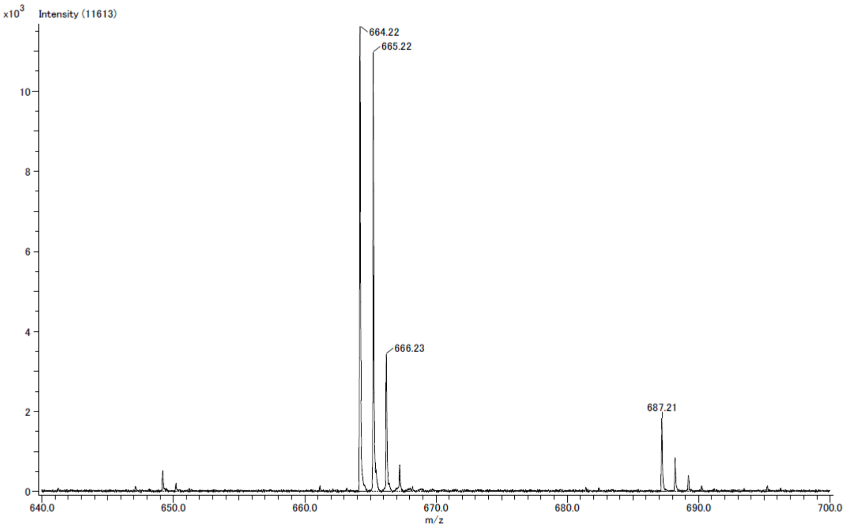

TOF-Mass of ***SQ-2***

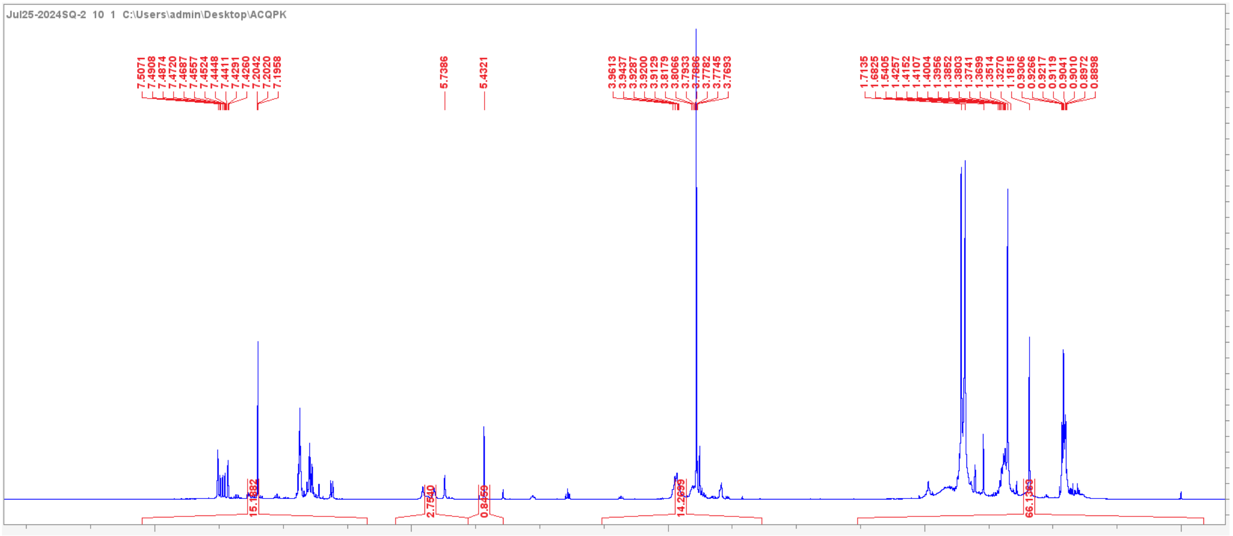

1H NMR of ***SQ-2***

S1.8. Synthesis of Unsymmetrical Dye SQ-3

A total of 1.46 mmol (500mg) of the intermediate (6*) and hydrolyzed semi-squaraine dye intermediate (4) were dissolved in 30 mL toluene/butanol (1:1) in a round-bottom flask fitted with a condenser. The reaction mixture was then refluxed for 8 hours followed by solvent removal with a rotary evaporator. The crude dye was then subjected to flash column chromatography using $CHCl_3$/MeOH (9:1) as the eluting solvent, giving titled dye SQ-1 as a blue solid in 85% yield.

TOF-Mass (measured 554.287 [M+H]+; 555.26 calculated). 1H NMR (500 MHz,$CDCl_3$): δ/ppm = 0.93-0.89 (t, CH_3) ; 0.97 (s, CH_3) ; 1.42-1.34 (m, CH_2) ; 1.70 (s,CH_3); 2.82-2.79 (t, CH_2); 3.77 (s, O-CH_3); 3.95-3.92 (t, CH_2) ; 5.85 (s, CH methylene); 6.77 (s, CH methylene); 6.79 (d, CH benzene); 6.88(s, CH methylene);(7.08-7.05 (t, CH benzene); 7.03- 7.00 (t, CH benzene); 7.19 (s, CH benzene); 7.23 (d, CH benzene); 7.28-7.26 (d, CH benzene) confirms the identity of the synthesized product.

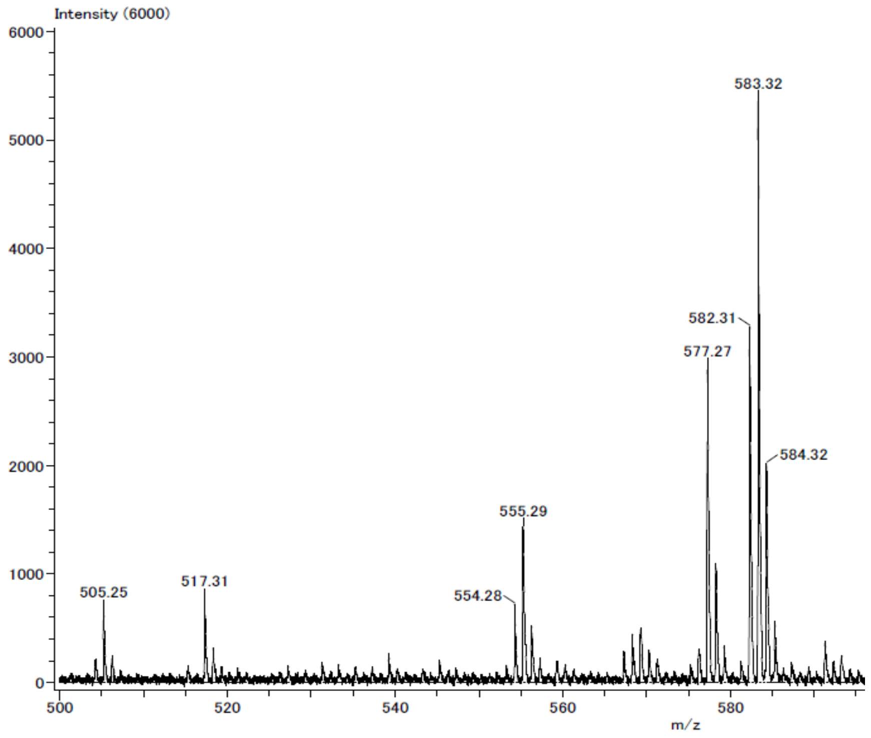

TOF-MS of ***SQ-3***

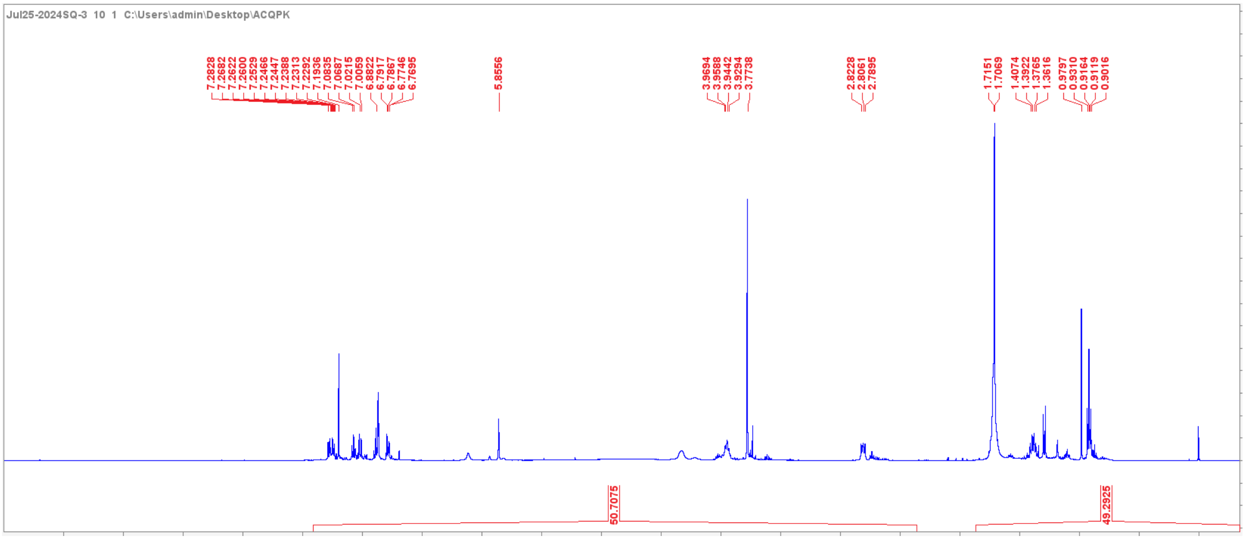

1H NMR of ***SQ-3***

S3. Solid Phase Synthesis of Peptide Sequence

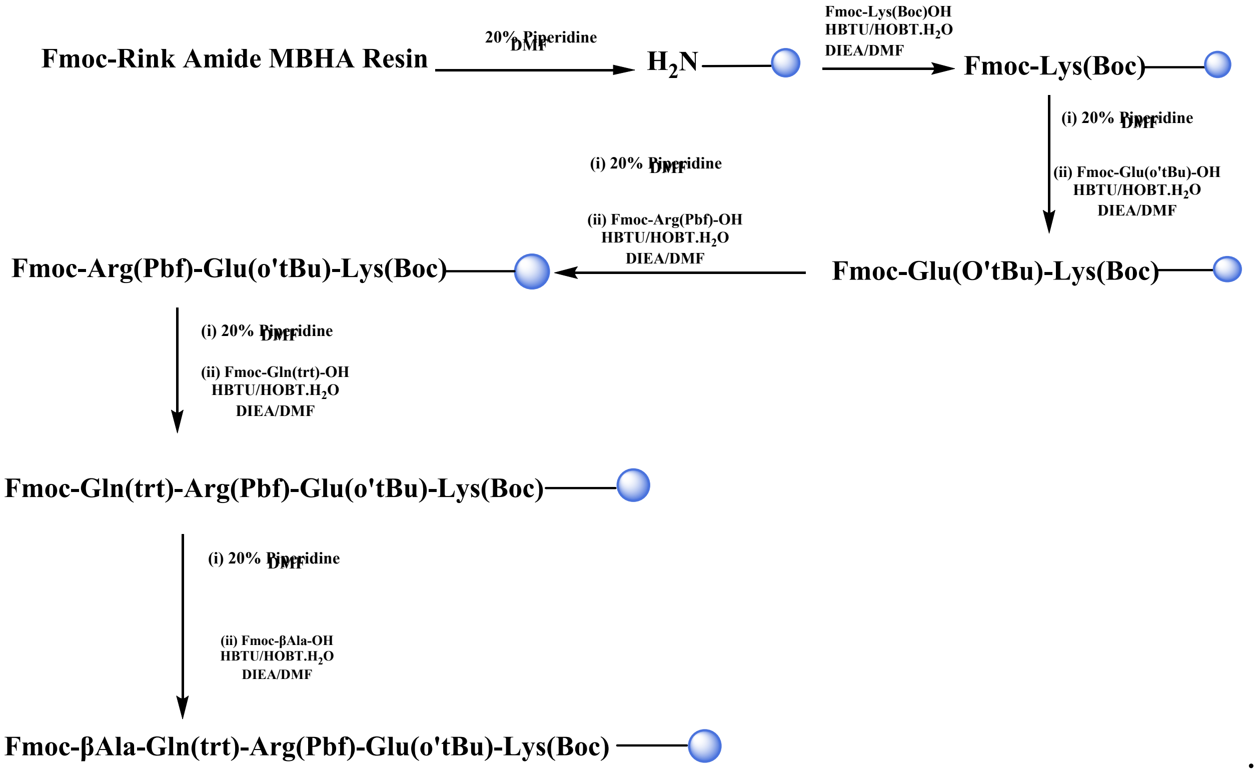

Scheme S3. Scheme for the synthesis of peptide sequence

Fmoc-Lys (Boc)OH was loaded onto Rink Amide MBHA resin (substitution 0.41 mmol/g resin) using Fmoc/piperidine strategies on a 0.432 mmol scale. HBTU and HOBT.H_2O were used as activating agents. Subsequently, Fmoc-Glu(o'tBu)-OH, Fmoc-Arg (Pbf)-OH, Fmoc-Gln(trt)-OH, Fmoc-Ala-OH, and Fmoc-β-Ala-OH were anchored. A small amount of the corresponding resin-supported peptide was subjected to TFA/Triisopropylsilane/H_2O = 95:2.5:2.5 and the resin-supported peptide was cleaved from the resin in the cleavage cocktail. The peptide was precipitated by ether in an ice bath. The purified substrate was analyzed by (TOF-MS). TOF Mass (measured 852.42 [M]+; 852.44 calculated) confirms the identity of the synthesized product.

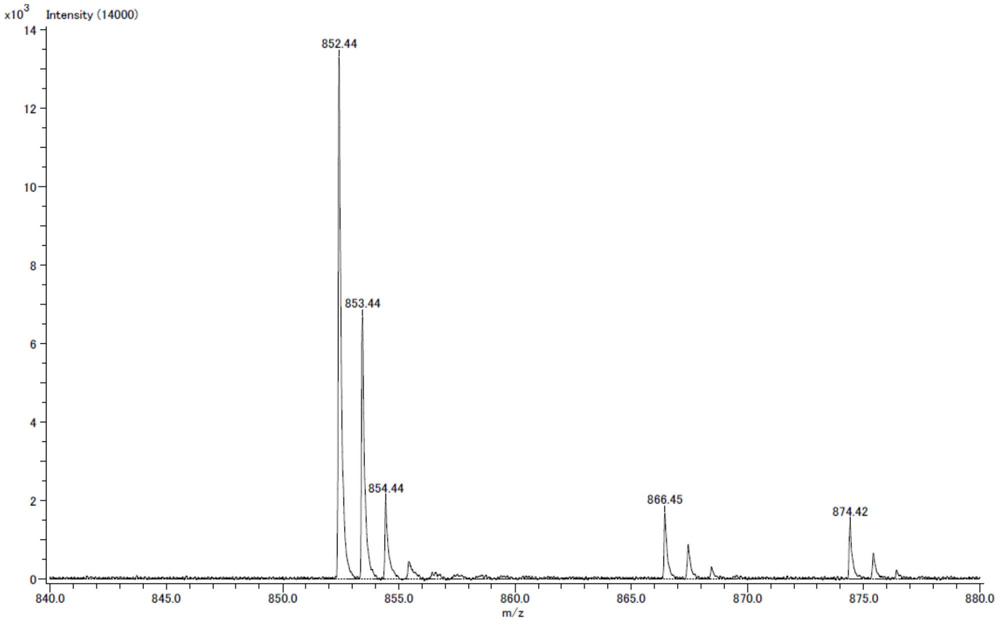

TOF-Mass of ***peptide***

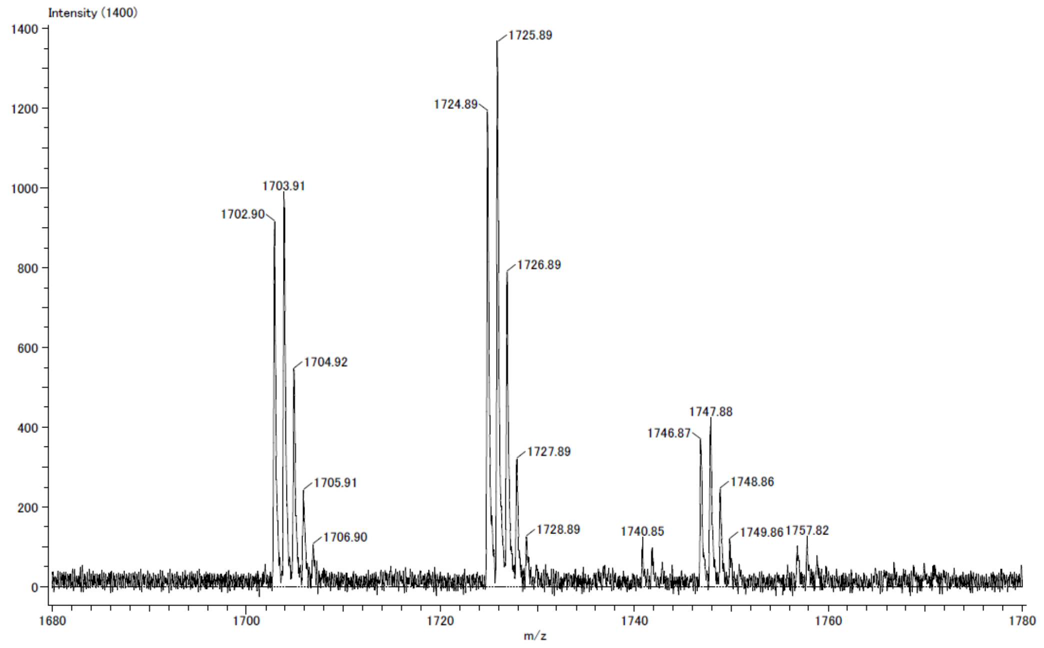

TOF-Mass of ***SQ-3 PC***

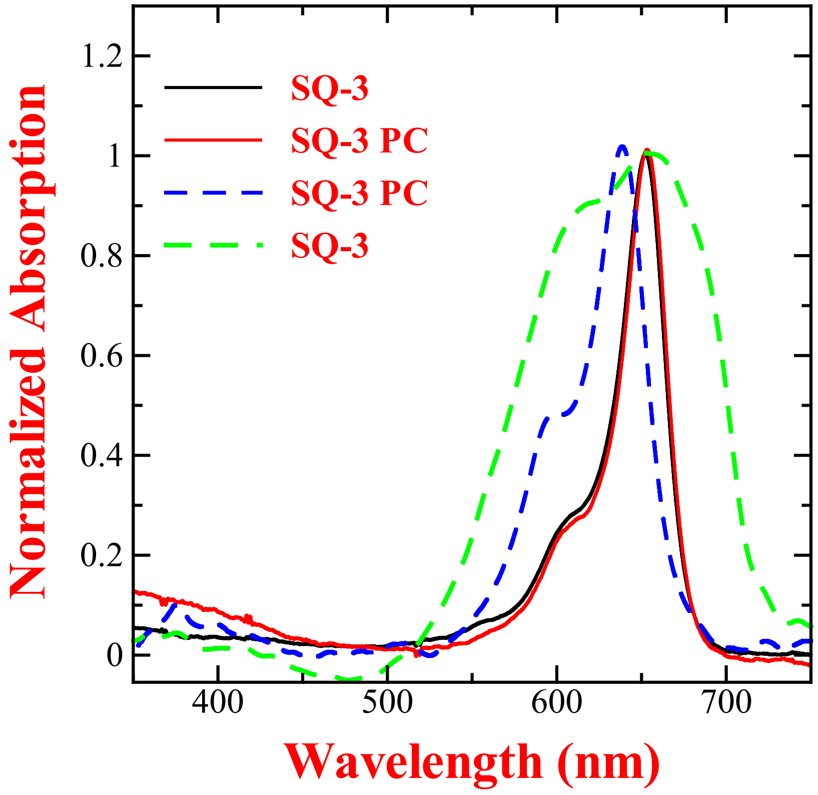

Figure S1. Normalized absorption spectra of SQ-3 and SQ-3 PC in DMSO (solid line) and H_2O (2% DMSO) (dash line) at a concentration of 5 μM.

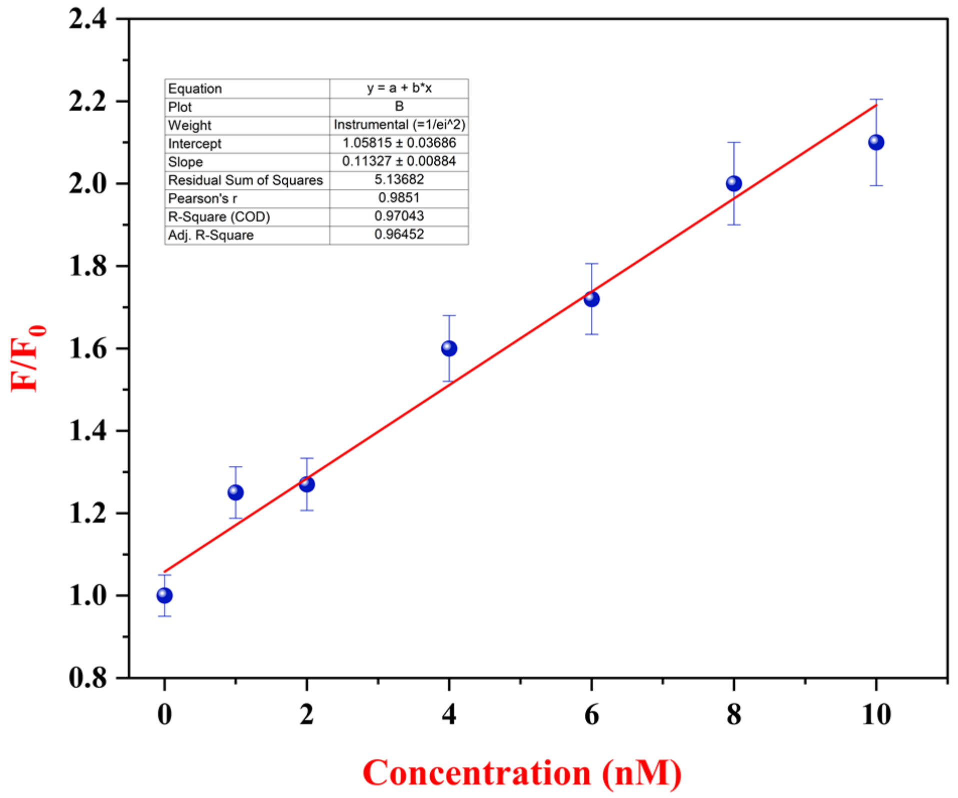

Figure S2. Linear correction curve of ratio of fluorescence intensity against trypsin concentration (0 nM to 10 nM).

Table S1. Comparison of different methods for the determination of trypsin.

Method	Sensitivity (LOD)	Specificity	Linear Range	Practical Applications	Ref.
Fluorometry/Ag NCs	2.55 nM	High	30 – 174 nM	Bioanalysis for trypsin activity assay	[1]
Fluorometry/AgInZnS QDs	1.74 nM	High	4 – 174 nM	Imaging of living cells and	[2]

				localization of lysosomes	
Glutathione-stabilized gold nanoclusters	3.478 nM	High	8 nM – 4348 nM	Point-of-care detection	[3]
Magnetic/photoluminescence bifunctional nanohybrid	10.87 nM	High	22 nM – 1304 nM	Determination of trypsin in human urines	[4]
**NIR Fluorescence/ SQ-3 PC**	**1.03nM**	**High**	**0-75 nM**	**In vitro assays, the potential for in vivo imaging**	**This work**
